# Takotsubo Cardiomyopathy: Medical and Psychiatric Aspects. Role of Psychotropic Medications in the Treatment of Adults with “Broken Heart” Syndrome

**DOI:** 10.7759/cureus.5177

**Published:** 2019-07-19

**Authors:** Valeriy Zvonarev

**Affiliations:** 1 School of Behavioral Sciences, California Southern University, Costa Mesa, USA

**Keywords:** antidepressants, selective serotonin reuptake inhibitors (ssri), psychological distress, trauma, catecholamines, takotsubo cardiomyopathy, tca, snri, acute coronary syndrome (acs)

## Abstract

Takotsubo cardiomyopathy (TTC) is reversible stress-induced cardiomyopathy featuring symptoms of acute myocardial infarction without significant coronary artery abnormalities. TTC is frequently precipitated by stressful emotional events but it also has been reported as a result of substance withdrawal, non-cardiac events, and dangerous drug-to-drug interaction. The plasma levels of both epinephrine and norepinephrine were significantly elevated in TTC patients, suggesting that elevated catecholamine levels might be the main contributing factor. However, the mechanisms underlying susceptibility to development and recurrence are not completely understood.

It has been suggested that even a therapeutic dose of antidepressant could be a cause of drug-induced tachycardia and TTC. Moreover, some cases have been reported in which the development of TTC was associated with the serotonin syndrome, neuroleptic malignant syndrome, and similar fatal consequences.

The aim of this article is to explore the association between underlying psychiatric disorders and TTC and to determine the role of various psychotropic medications in the progression of stress-induced cardiomyopathy. This article also notes and discusses the current theories underlying the pathophysiology of TTC. This review suggests a serious side effect of antidepressants, and to avoid life-threatening cardiovascular events, such as TTC, for patients with affective and anxiety disorders, prior screening for cardiovascular conditions by ECG with close monitoring might be necessary.

## Introduction and background

Takotsubo cardiomyopathy (TTC) is characterized by transient apical and mid-ventricular left ventricular (LV) dysfunction in the absence of significant coronary artery disease. It is triggered by emotional or physical stress. It shows symptoms that are similar to those of myocardial infarction (MI), however, it does not occur as a result of any underlying cardiovascular condition. It is a reversible heart condition, which occurs almost entirely in postmenopausal women. The condition causes waning of the left ventricle, the main pumping chamber of the heart. In a setting of depressed distal and apical LV function, there is compensatory hyperkinesis of basal walls. It was suggested that TTC is associated with extreme emotional and physical stress. However, the pathophysiology of this condition has not been fully elucidated. According to preliminary data, catecholamines released from adrenal chromaffin cells and norepinephrine released from sympathetic nerve terminals are significantly increased in the acute phase of TTC. Thyrotoxicosis, pheochromocytoma, and several neuropsychiatric diseases are medical conditions associated with TTC. This article will discuss these issues and shed some light on the pathogenesis of this syndrome and the role of various psychotropic medications in the treatment of adults with TTC.

Methods

We conducted a systematic literature review by identifying, critically evaluating, and integrating the findings of all relevant studies addressing the following research aspects:

1. The pathophysiological role of catecholamines and underlying psychiatric disorders in the development of Takotsubo cardiomyopathy

2. The morbid effect of antidepressant drug therapy in patients with TTC

We also discuss the clinical presentation of TTC, review relevant causes, and discuss the use of various psychiatric medications in patients who are at increased risk of developing this cardiomyopathy. The following criteria were used to select articles for the literature review based on the research question:

1. Included studies must have compared certain treatments (selective serotonin reuptake inhibitor (SSRI), selective norepinephrine reuptake inhibitor (SNRI), tricyclic antidepressant (TCA), etc.)

2. Included studies used an observational and experimental design

3. Included studies must have been published in the last 10-15 years

4. Studies that used a quantitative methodology preferred

5. Comprehensive sources and peer-review journals only

6. Included studies must have been stored in the following databases: Cumulative Index to Nursing and Allied Health Literature (CINAHL), Cochrane Library, Education Resources Information Center (ERIC), PsycINFO, PubMed/MEDLINE

The search process was documented in enough detail throughout the process to ensure that it can be reported correctly in the review. In accordance with the criteria mentioned above, three (3) comprehensive searches were conducted between May 06, 2019, and June 14, 2019. For the topic selected earlier, the keywords are cardiomyopathy, takotsubo, depression, anxiety, epinephrine, norepinephrine, and antidepressants. Approximately 100 results were retrieved. The following years were selected: 2003-2018. Several filters were applied. A comprehensive evaluation of each study quantity was conducted.

Background

Takotsubo Cardiomyopathy: Definition, Epidemiology, and Statistics

TTC is a cardiac condition that causes an unexpected and momentary waning of the heart’s muscular section [1]. The weakening of often triggered by emotional stress from situations like emotional break-ups, the demise of a loved one, or excessive anxiety. It has been recognized as a leading cause of ventricular ruptures, acute heart failure, and lethal ventricular arrhythmias [2]. The name of the condition is derived from the Japanese word takotsubo, which is an octopus trap. The nomenclature relates to the similar shapes of the octopus trap and the left ventricle.

Before the condition was officially named tako-tsubo, it existed, but the syndrome was diagnosed differently in different geographical areas. It has been recognized as a severe heart condition, with similar clinical appearance to other coronary events [2].

The first case was reported in Japan in 1990. Since the Japanese term was introduced, the condition has increasingly been noted in the majority of countries. Unlike other cardiomyopathy conditions, TTC is not known to be inherited.

The prevalence of Takotsubo cardiomyopathy has been reported in 2% of patients with apparent acute coronary syndrome and 10% of female patients. It is seen in persons of any age group, however, it is more common in postmenopausal females than in males. Studies show that 85%-90% of patients with the condition are females ranging in age from approximately 65 to 70 years old. More specifically, one study shows that 89.8% of recent cases of the conditions were women with a mean age of 67 years. Despite these figures, TTC has been reported in people of both genders and from every age group [3]. In men, the predisposing factor is most likely physical stress or severe medical illness.

In the past, the prevalence of TTC was probably underestimated due to widespread ignorance of the syndrome. Conversely, with the growing consciousness about the disease and an overall increase in early access to angiography, the condition is not identified more frequently. Studies and health reports have identified an increased incidence of TTC. There has also been an increased hospitalization rate of individuals with TTC [4].

**Figure 1:**
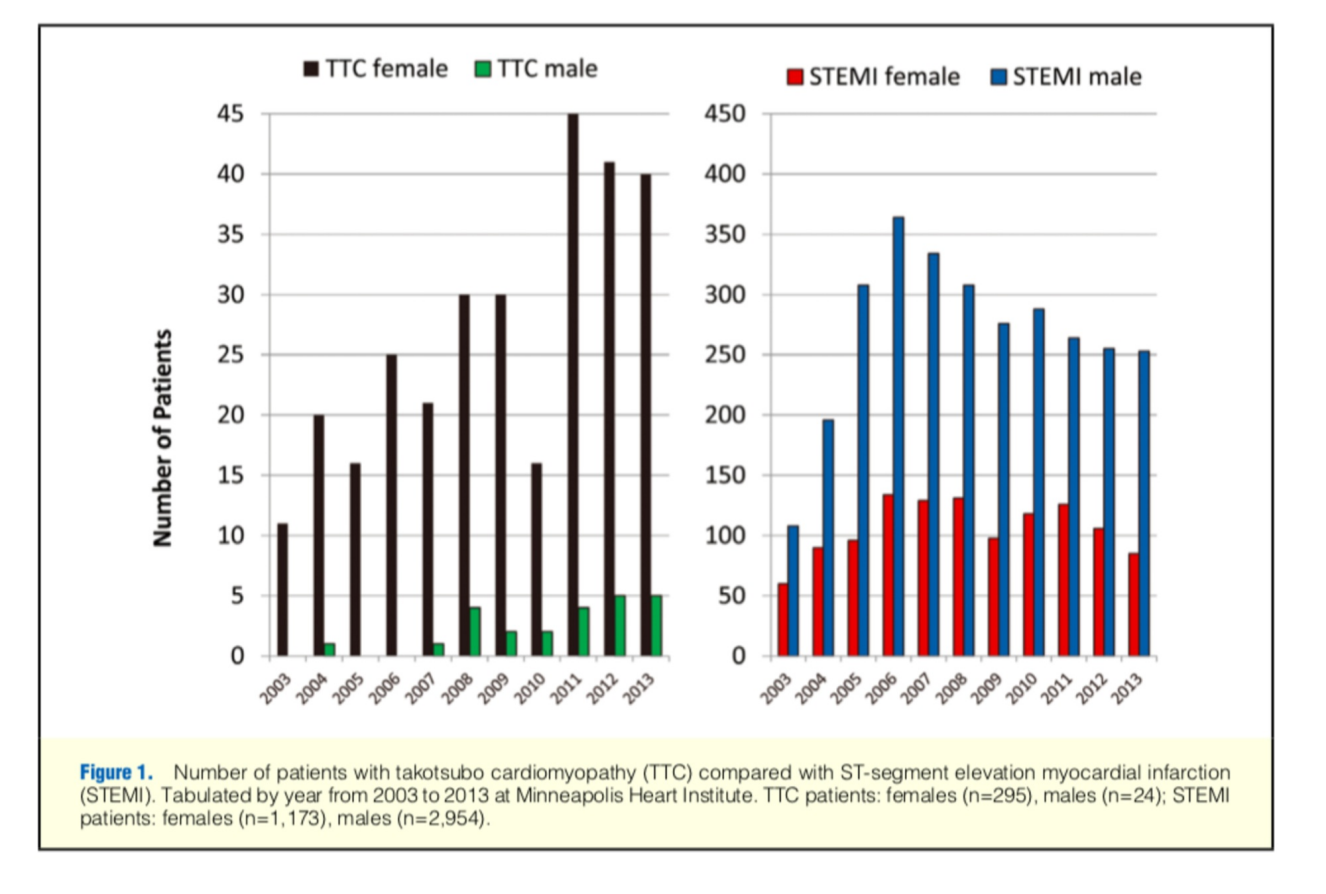
Number of patients with TTC compared with STEMI TTC: Takotsubo cardiomyopathy; STEMI: ST-segment elevation myocardial infarction

The number of documented cases has progressively increased since 2001 (Figure 1) [5].

**Figure 2:**
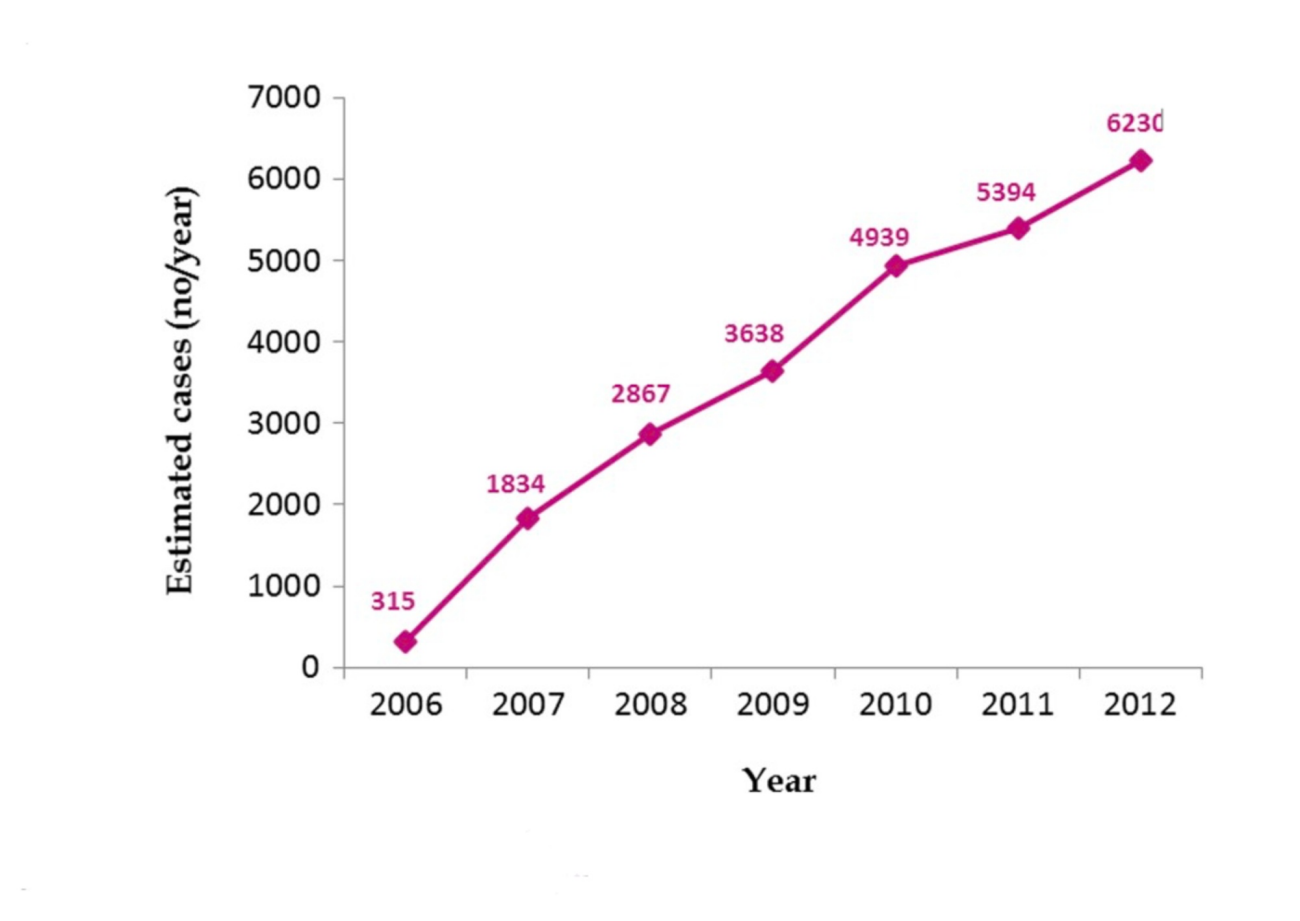
Trends in reported incidence of Takotsubo syndrome from 2006 to 2012 Modified from a previously published table by Minhas AS et al.

Writers reported a significant increase in the incidence of TTC from 2006 to 2012. In this study, the incidence of TTC increased almost 20 times during the time period [6]. Results are summarized in Figure 2.

Though the first case was reported in Japan, today, TTC is geographically widespread and has been reported in all six continents [7]. It has been reported in more than 50 countries with little variation in its clinical expression. Although there have not been large comparative studies, preliminary studies show that cases are reported in many races but are less common among African Americans and Hispanics [3]. A study was also not able to identify significant differences in clinical manifestations between Caucasian and Asian patients with TTC. Existing data suggest a temporal pattern of occurrence, as it has been associated with a circadian effect, with peaks in the morning and lows in the evening. This is associated with the morning flow of stress hormones and the experience of stressful events at a particular time of the day.

Classification of Takotsubo Cardiomyopathy

Based on wall motion abnormalities, TTC could be classified as:

Classical type: apical ballooning

Reverse type: hyperdynamic apex and akinesia of the base of the left ventricular wall

Midventricular type: involves the mid-LV wall, sparing the base and the apex

Localized type: any other segmental ballooning when Takotsubo-like LV dysfunction is present

There are five subtypes of TTC [1]. All types are summarized in Table 1.

**Table 1:** Types of Takotsubo cardiomyopathy

Class I	Takotsubo syndrome related to emotional stress
Class II	Takotsubo syndrome related to physical stress
Class IIa	Takotsubo syndrome secondary to physical activities, medical conditions, or procedures
Class IIb	Takotsubo syndrome secondary to neurological disorders
Class III	Takotsubo syndrome without an identifiable triggering factor

**Figure 3:**
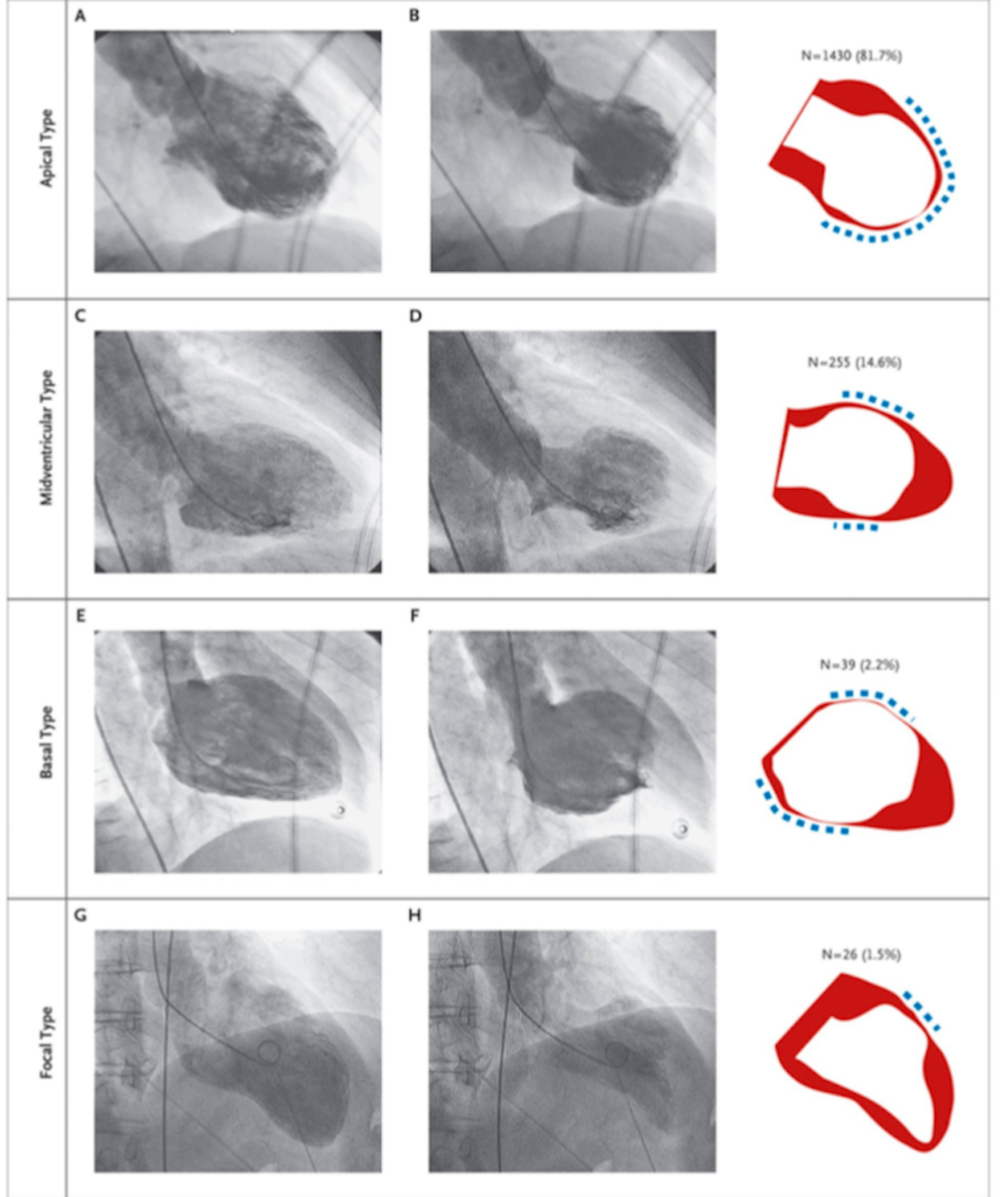
Various types of Takotsubo cardiomyopathy The four types of Takotsubo cardiomyopathy. Apical type (panels A and B), midventricular type (panels C and D), the basal type (panels E and F), and the focal type (panels G and H). Source: Templin C, Ghadri JR, Diekmann J, Napp LC, Bataiosu DR, Jaguszewski M, et al. Clinical features and outcomes of Takotsubo (stress) cardiomyopathy. N Engl J Med. 2015;373 (10):929–938.

Among the 1750 study participants, the most common type of TTC was the apical type (in 81.7% of patients) (A and B), followed by the midventricular type (in 14.6% of patients) (C and D), the basal type (in 2.2% of patients) (E and F), and the focal type (in 1.5% of patients) (G and H). In the right column, the wall-motion pattern that was recorded with each type of the syndrome is shown, with red indicating diastole, white indicating systole, and the dashed line indicating the location of the wall-motion pattern (Figure 3) [2].

Risk Factors of Takotsubo Cardiomyopathy

The development of Takotsubo cardiomyopathy is often preceded with significant physical or emotional stress or neurologic injury. Common stressors include news of the death of a loved one, financial issues, natural disasters, legal problems, motor vehicle accidents, long stays in intensive care units, a recent medical diagnosis, and substance withdrawal from substance abuse, among others [8]. TTC is also seen with seizure activity. Though the clinical presentation is often similar to that of other cardiac conditions, it can be differentiated through ventriculography used together with echocardiography. In most patients, it is clear that physical stressors are the most common causes, as opposed to emotional stressors, while a significant portion of patients did not have clear triggers. Most affected people also psychiatric or neurological conditions [9]. The risk factors are summarized in Table 2.

**Table 2:** Risk factors of Takotsubo cardiomyopathy

Emotional risk factors	Physical risk factors	Medical risk factors	Surgery-related and other risk factors
Death of a loved one (including pets)	Physical exhaustion (triathlon, sexual, gym): males	Opiate withdrawal, alcohol consumption, smoking	Hysterectomy
Surprise party	Cold exposure	Thyrotoxicosis, pheochromocytoma	Cholecystectomy
Family member being arrested	Trauma	Asthma attack, pneumothorax	Fear of procedure
Fear of choking Fear of catastrophic medical diagnosis	Severe pain and chronic pain syndromes	Ventricular fibrillation, Stress echo with dobutamine (cardiac stress test)	Other non-cardiac invasive procedures
Robbery or any kind of physical/emotional abuse		Psychiatric disorders (anxiety and mood disorders specifically)	
Public speaking, court appearance or confrontational argument		Neurological disorders (epilepsy, cerebrovascular accident)	

Etiology and Pathogenesis of Takotsubo Syndrome

There are several theories that have been proposed and are under investigation. Such theories include acute cardiac syndrome with reperfusion injury, multivessel coronary artery spasm, impairment cardiac microvascular function, and impairment of the metabolism of fatty acids, among others. The etiology can be explained as an acute and reversible left ventricular dysfunction, which occurs in patients without previous recorded coronary illness. The syndrome is thought to be caused by exposure to catecholamines facilitated through inflated sympathetic stimulation [10]. TTC happens in patients with critical illness but without any other coronary diseases. This variant of Takotsubo happens in patients who are in their usual health state, often subsequent to undergoing physical or emotional stress.

Pathogenesis

The pathogenesis of Takotsubo cardiomyopathy has not been entirely understood. It has not been identified why the condition disproportionately affects post-menopausal women or even why it primarily affects the mid cavity and the apex of the left ventricle [11]. A study comparing the diastolic and systolic functioning of patients' left ventricle for those with broken heart syndrome and patients suffering from severe MI points to striking conclusions. The initial systolic function is found to be similar or worse among those with broken heart syndrome as compared to those with MI; on the other hand, the diastolic function could be the same or healthier among those having TTC [11]. Some of the proposed mechanisms include microvascular dysfunction, presence of excess catecholamine, and coronary artery spasm. In some patients, dynamic mid-cavity or obstruction of the outflow tract of the left ventricle have also been recorded, which can result in apical dysfunction.

The underlying pathophysiology of the disease has not been fully understood, however, it is argued that it occurs as a result of stress and the subsequent release of catecholamines. The condition is reportedly precipitated by both emotional and physical stressors [12]. Another factor that could be playing an active role in the development of TTC is the adrenal-cardiac axis, which is involved by the antagonistic remodeling process with protracted cardiac failure. In addition, it can contribute to the parthenogenesis of the condition. Some studies have shown that increased circulation of catecholamine plays a vital role in the parthenogenesis of the illness. According to recent data, the plasma levels of epinephrine and norepinephrine are increased, even higher than in patients with similar heart failure (HF). Though the release of catecholamine is supposed to contribute to transient myocardial stunning, it has not been fully understood why the apex is, in most instances, involved selectively when basal contraction is spared [12]. Studies have recently led to more insight on the likely causes of the selective involvement of the myocardium on the ventricle apex and the discrepancy in distribution within the cardiac adrenoreceptors. Once catecholamine is released, it results in basal hyperkinesis in the left ventricle and the resulting mitral reduction, motion in the systolic anterior, and increase in intraventricular pressure cause apical ballooning.

In general, the catecholamine toxicity theory can be summarized in three steps:

1) Apical-basal density of β-adrenergic receptors (β-ARs) and sympathetic innervation in human LV, where the apex contains the highest β-AR and the lowest sympathetic nerve density. The presence of ventricular β-AR results in increased apical sensitivity to epinephrine [13].

2) High concentrations of epinephrine can cause a negative inotropic impact and trigger a switch from Gs (stimulatory) protein to Gi (inhibitory) protein signaling through the β2AR. This negative inotropic effect is strongest in the cardiac apex where the density of β-ARs is highest.

3) LV dysfunction in TTC is associated with an oxidative stress response to an excess of catecholamines.

Pheochromocytoma and massive cerebral vascular accidents are linked to transient myocardial dysfunction, which can result in the catecholamine pathophysiology of TTC [12]. Patients who have experienced pheochromocytoma are likely to experience catecholamine-related cardiomyopathy due to remodeling and cardiac toxicity. In addition, experiencing an extreme neurologic injury like hemorrhage in the subarachnoid can be linked to abnormalities in the electrocardiogram as well as systolic dysfunction of the left ventricle, a condition known as neurogenic stunned myocardium.

**Figure 4:**
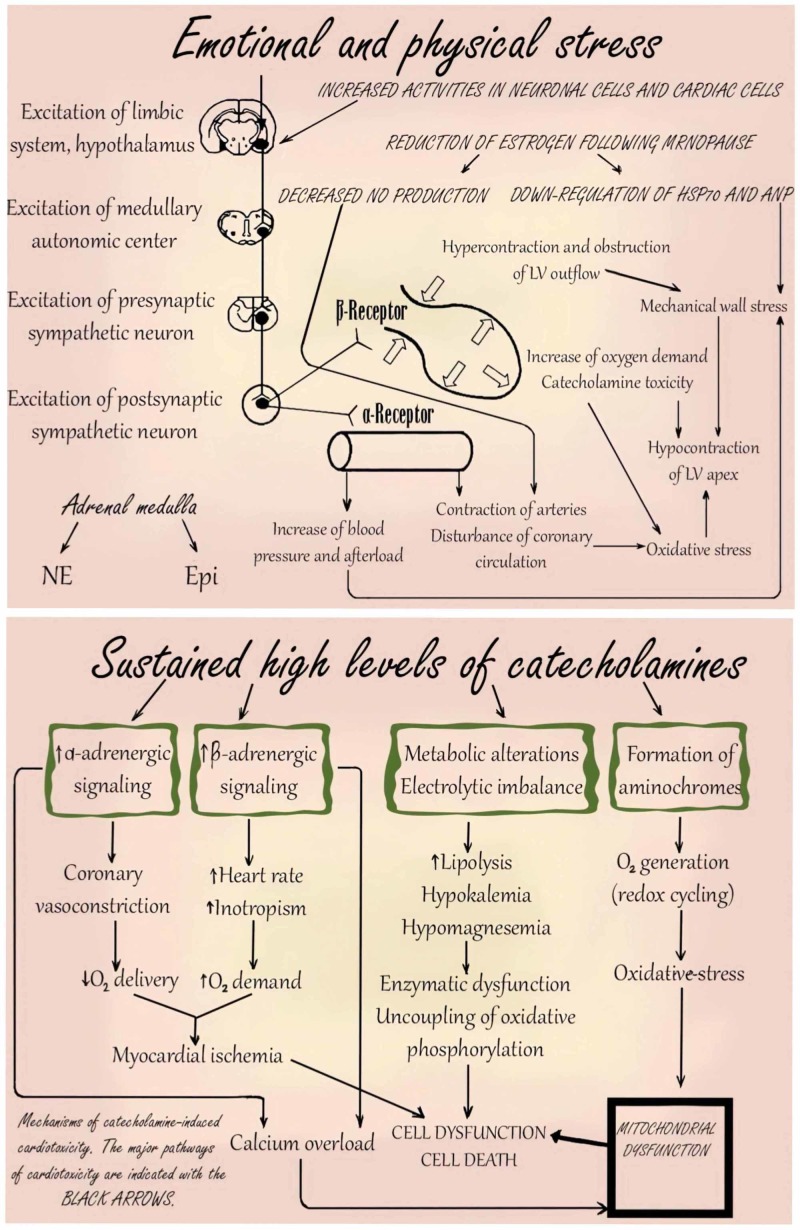
Pathogenesis of Takotsubo cardiomyopathy Adapted from Akashi Y, Goldstein D, Barbaro G, Ueyama T: Takotsubo cardiomyopathy: a new form of acute, reversible heart failure. Circulation. 2008, 118:2754-2762.© LV: left ventricle; Epi: epinephrine; NE: noradrenaline; HSP70: heat shock protein 70; ANP: atrial natriuretic peptide; NO: nitric oxide

Besides, there is also another mechanism of action that involves vasospasm of the coronary artery and the coronary microvascular dysfunction. Most patients studied show evidence of coronary spasms. In a test where spasms in multiple coronary arteries were induced through provocative testing, it is revealed that weakened coronary microcirculation contributes to the pathophysiology of Takotsubo cardiomyopathy. In the past, there have also been some proposals that the pathophysiology may be linked to a halted case of MI. There is a proposal that a frontal infarct having an extended frontal artery down on the left can cause preferential hypokinesis at the apex. In a study modeling this suggestion against a control group, it was not a common occurrence to find an extended frontal descending artery enfolding about the peak when compared to the control group. A recent study presented the pathogenesis of TTC as shown in Figure 4 [14].

Clinical Presentation and Diagnosis 

The clinical presentations of the patients who are finally diagnosed with TTC cannot be distinguished from those with acute coronary syndrome. The most likely symptoms include nausea, chest pain, syncope, dyspnea, palpitations, and cardiogenic shock [15]. One unique feature of the condition is the occurrence of either physical or emotional trigger events, which are reported in about two-thirds of the patients. As opposed to acute coronary syndrome, which most often occurs in the morning hours, Takotsubo cardiomyopathy often occurs in the afternoons when there are more chances of experiencing the triggers [12]. A significant portion of the patients with broken heart syndrome show the symptoms of cardiogenic shock, abrupt cardiac arrest, and tachyarrhythmia [16]. Some patients may also develop signs and symptoms of stroke or transient ischemic attack. Such patients are most likely to have ST-segment elevation (usually anterior precordial leads) while ST-segment depression is less likely among patients with the condition. The findings can also include T wave inversion, abnormalities in the Q waves, QT interval prolongation, among other abnormalities [15]. Most patients will have elevated levels of serum cardiac troponin, brain natriuretic peptide, with the creatine kinase remaining normal or being mildly elevated. The absence of clear diagnostic criteria for TTC has been known to be an important source of diagnostic unreliability for decades. Mayo Clinic has listed four total diagnostic criteria that are used in the diagnosis of Takotsubo cardiomyopathy and summarized in Table 3.

**Table 3:** Mayo Clinic criteria to diagnose TTC LV: left ventricle; CAD: coronary artery disease; ECG: electrocardiography; TTC: Takotsubo cardiomyopathy; ST: ST-segment of ECG; RWMA: regional wall motion akinesia

1	Typical LV contraction pattern: transient hypokinesia, akinesis or dyskinesia in the LV mid-segments with or without apical involvement accompanied with hypercontraction in the basal segments; RWMA that extend beyond a single coronary artery vascular distribution; a stressful trigger is usually but not always present;
2	Absence of obstructive CAD or angiographic evidence of acute plaque rupture;
3	Newly developed ECG abnormalities (ST-segment elevation and/or T-wave inversion) or modest elevation in cardiac troponin;
4	Absence of recent head trauma, intracranial hemorrhage, pheochromocytoma, myocarditis or hypertrophic cardiomyopathy

**Figure 5:**
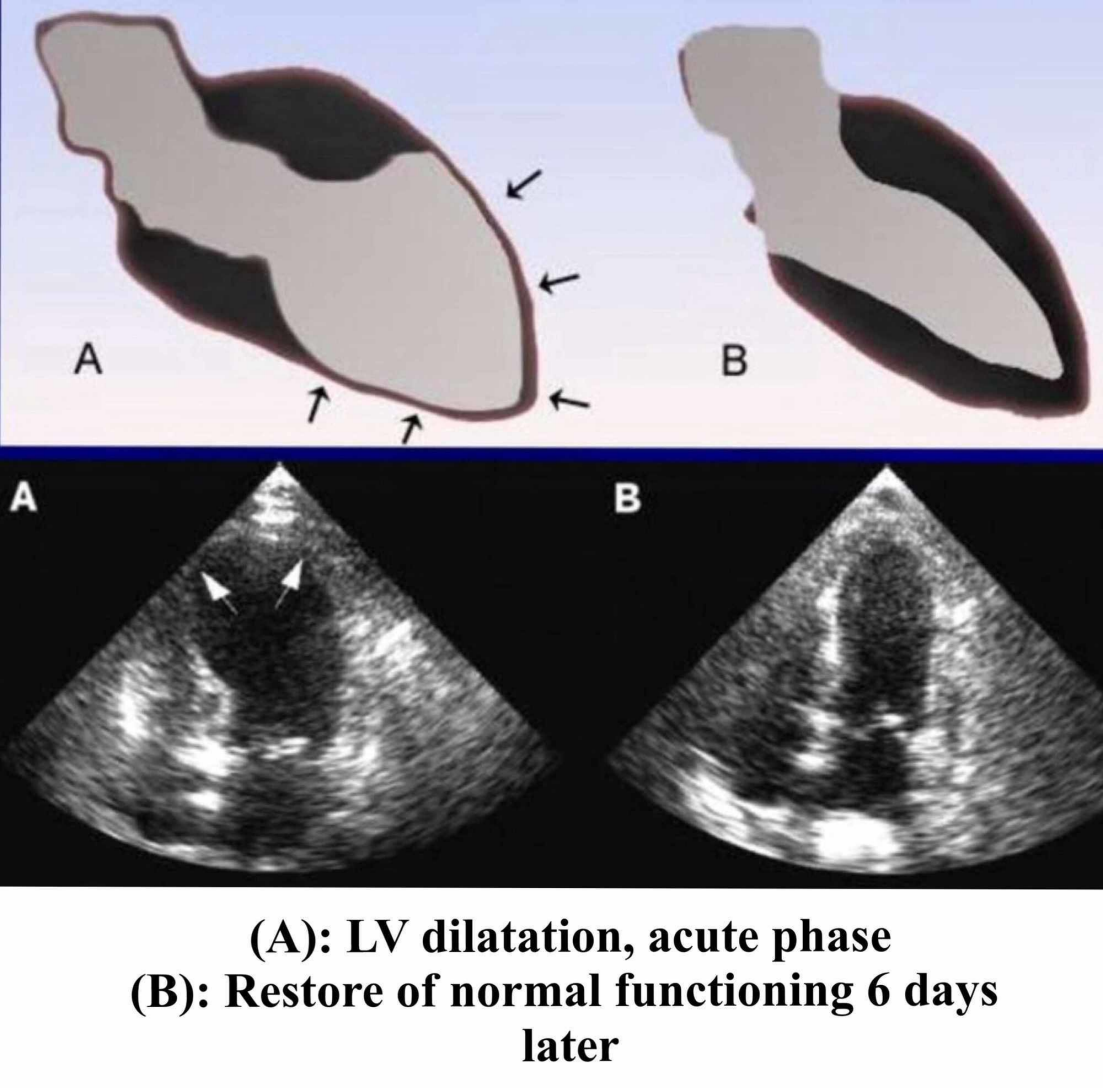
Echocardiographic changes in TTC TTC: Takotsubo cardiomyopathy

Several tests can be used to confirm the diagnosis [17]. LV ventriculogram and echocardiography can both be used to visualize apical ballooning with dyskinesis of the apical one-half to two-thirds of the LV. The average LV ejection fraction (EF) range is 20% to 49%. Apical ballooning is described as the classical angiographic manifestation of Takotsubo. However, it has also been shown that dysfunction of the left ventricle not only includes apical ballooning but also different angiographic morphologies like mid-ventricular ballooning and, in rare instances, ballooning of other segments. “Atypical” ballooning of the middle or basal portions of the LV are much less common. Wall motion abnormalities typically involve the distribution of more than one coronary artery. Ventriculography and echocardiography also allow evaluation of the LV outflow tract obstruction. Figure 5 shows relevant changes and abnormalities. Cardiac catheterization reveals lack of flow-limiting coronary lesions or evidence of plaque rupture.

**Figure 6:**
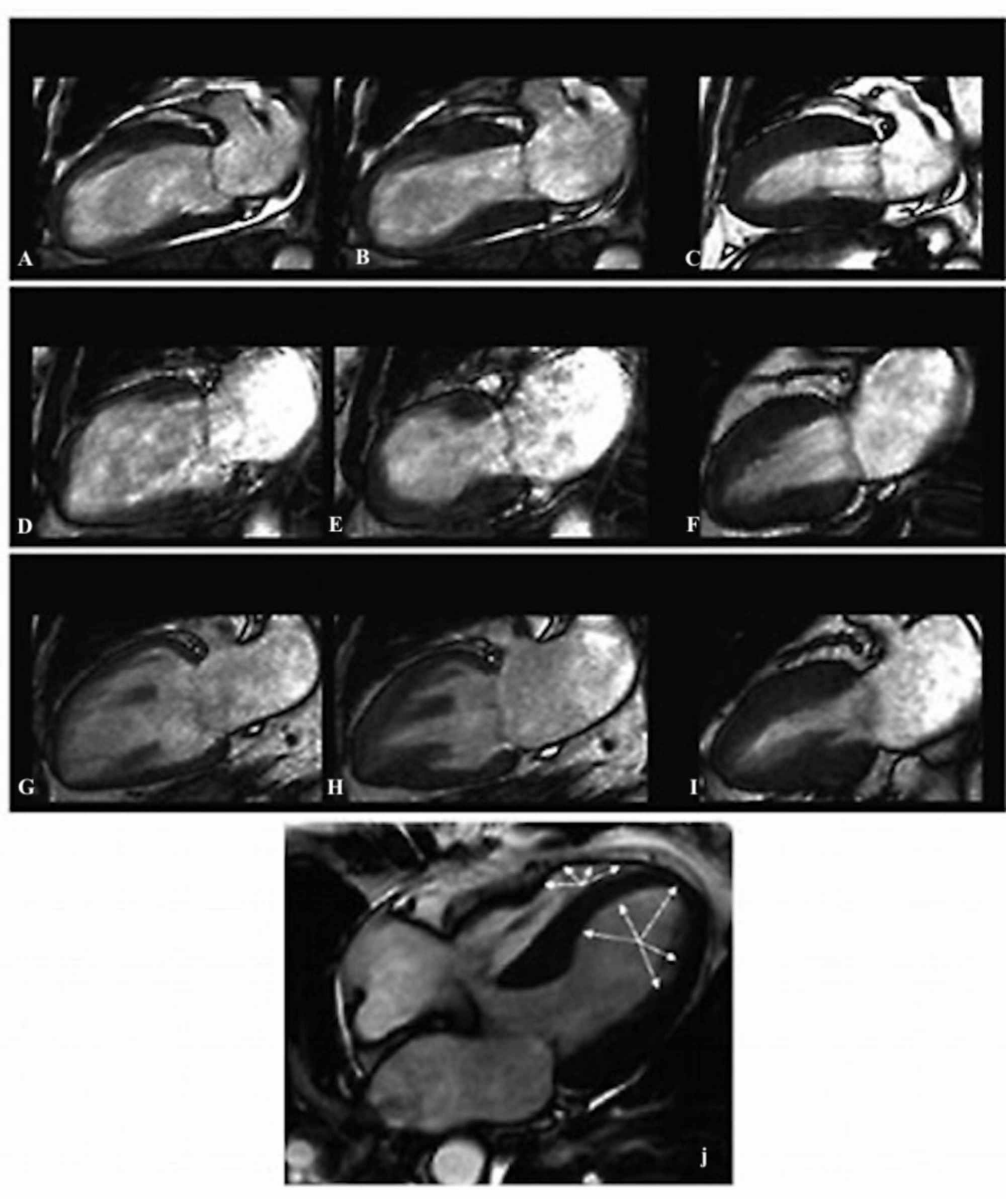
Assessment of TTC with CMR Typical apical ballooning in Takotsubo syndrome. Cine CMR 4-chamber view. Cardiac specific sequences are implemented in 1.5 T scanner. A, B, C: apical type of TTC; D, E, F: midventricular type of TTC; G, H, I: inverted type of TTC; J: biventricular type of TTC with areas of hypo/akinesia (white arrows); CMR: cardiovascular magnetic resonance; TTC: takotsubo cardiomyopathy

Cardiac magnetic resonance imaging (MRI) is prone to reveal ventricular edema that appears as high signal intensity with a diffuse or transmural distribution. Figure 6 shows all relevant findings. Ventricular edema is usually distributed in both the apical and mid planes of the LV. The area of edema usually manifests as dysfunction in the ventricular contractility observed with a cine MRI sequence [18].

## Review

Association of psychological disturbances and TTC: focus on psychopharmacology and psychological well-being

No clear evidence-based approach has been established to handle stress cardiomyopathy. This condition does not have treatment methods that can be used to prevent it from occurring, however, it is important for the patient to learn stress management, relaxation techniques, and problem-solving [11]. Healthcare providers usually recommend the typical pharmacologic therapy used for LV heart failure, including diuretics (if volume overloaded), angiotensin-converting enzyme (ACE) inhibitors, and beta-blockers [19]. Death due to TCM is a rare occurrence, however, about 20% of the patients experience heart failure [20].

People with the condition are often expected to stay in the hospital for three to seven days. Complete recovery always occurs within six months from the onset of the condition.

Given that psychological stress is associated with TTC, professionals recommend that patients learn healthy coping techniques to manage their stress.

The association of Takotsubo cardiomyopathy with depression and anxiety disorders is an underlying issue in the complicated framing of the condition. Depression levels in patients with TTC tend to fluctuate between 20.5% and 48% while the prevalence of anxiety disorders is between 26% and 56% [21]. The strong social inhibition and prominent depressive symptoms among TTC patients tend to be exacerbated with a high level of cortisol production, putting them at risk of uncontrolled and excessive worry.

Depression is associated with a number of hormonal changes:

1. Elevated cortisol levels and catecholamines

2. Nonsuppression of adrenocorticotropic hormone (ACTH) release in the dexamethasone suppression test

3. Chronically elevated levels of corticotropin-releasing hormone

**Figure 7:**
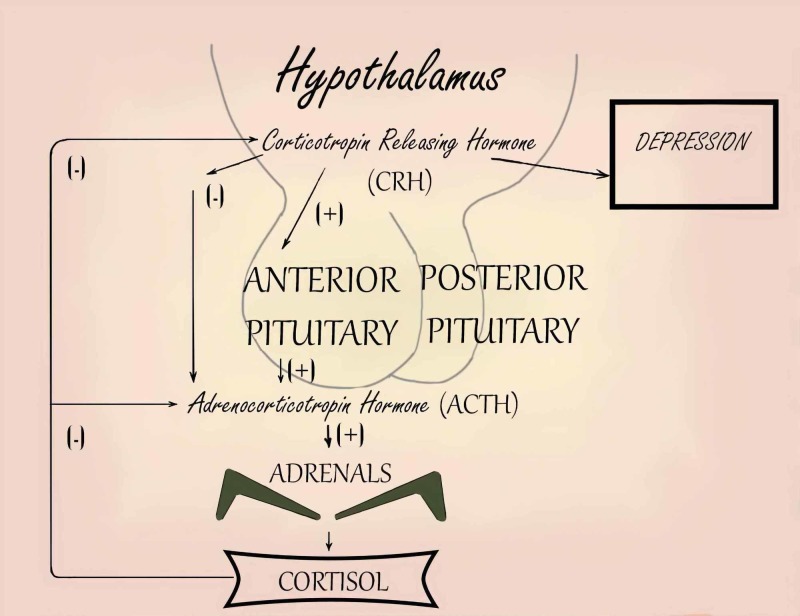
Association of depression with high levels of cortisol and catecholamines Adapted from Diapedia©. Retrieved June 23, 2019, from https://www.diapedia.org/associated-disorders/6104777426/depression-the-role-of-hpa-abnormalities CRH: corticotropin-releasing hormone; ACTH: adrenocorticotropic hormone

These changes are depicted in Figure 7.

Catecholamines activate the sympathetic nervous system and the hypothalamic-pituitary-adrenal (HPA) axis: increased secretion of dopamine and norepinephrine in the locus coeruleus induces secretion of CRF in the hypothalamus. Glutamate receptors are activated either by CRF or disinhibited by GABA oversecretion.

In general, TTC patients are likely to have a higher prevalence of anxiety and depressive disorders. In a case-control study conducted by Sancassiani et al., 19 consecutive patients (17 female and two male) with TTC compared to 76 controls without TTC were randomly selected from the database of a nationwide epidemiological study after matching (gender, age, and residence) by controls. It was noted that 17 patients with TTC (84.47% in total) reported experiencing at least one traumatic life event and depressive symptoms before being hospitalized in the cardiac unit [22]. Only major depressive disorder (MDD) showed higher frequencies in cases with a statistically significant difference (p=0.014) as well as at least one mood disorder diagnosis (MDD or bipolar disorder (BD)) (p=0.002). Moreover, it was found that the use of antidepressants was higher in the TTC group (15.79% vs 1.31%; p=0.030). As previously noted in the literature, however, the findings from this study did not confirm the association of TTC with anxiety syndromes [23]. Data from this study were based on theories that depressive mood disorders may lower patients vulnerability to stress. According to the sample used in this study, there was a high percentage of depressive disorders and life events in people with TTC. Sancassiani et al. (2018) concluded that antidepressant drugs have other effects, apart from the effect on depression but further studies need to be done to determine if there is a relationship between antidepressants use and TTC [23].

Table 4 includes and summarizes several studies reported the association of psychological disturbances and TTC.

**Table 4:** Association of TTC and psychiatric disorders TTC: Takotsubo cardiomyopathy; STEMI: ST-segment elevation myocardial infarction; FPI-R: The Freiburg Personality Inventory, Revised; SCL-90R: The Symptom Checklist-90-R (SCL-90-R); TS: Takotsubo syndrome; ACS: acute coronary syndrome; DS14: Type D Scale-14; MI: myocardial infarction; AMI: acute myocardial infarction; PGWB: Psychological General Well-Being index; ST: ST-segment of electrocardiogram; MDD: major depressive disorder

Authors	Description	Results
Kastaun, et al. 2014 [24]	Case-control stunt, 19 TTC, 20 STEMI, 20 healthy controls. FPI-R scale, SCL-90R checklist used.	Higher percentage of anxiety disorders and greater emotional lability in TS. 39% TTC vs. 5% STEMI
Deshmukh et al.2010 [25]	Cross-sectional study, 6837 TTC cases described and analyzed. ICD-9CM coding system used.	Anxiety was associated with higher rates of developing TTC.
Summers, et al. 2010 [26]	Case-control study, 25 TTC, 25 STEMI, 50 healthy controls. Medical and psychiatric records analyzed.	Anxiety: 56 % TTC vs. 12% STEMI; Depression: 48% TTC vs 28% STEMI. Anxiety or depression: 68% TTC vs 36% STEMI vs 30% health controls.
Compare et al. 2013 [27]	Case-control study, 37 TTC cases experienced emotional stress; 37 acute MI experienced emotional triggering; 37 TS w/o emotional stress. DS14 scale used.	Type D: 76% TTC emotional stress vs. 43% ST w/o emotional stress vs. 32% acute MI
Del Pace, et al. 2011 [28]	Case-control study, 50 TTC cases, 50 STEMI cases	High-anxiety trait: 60 % TS vs 52% STEMI
Delmas, et al. 2013 [29]	Case-control study, 45 TTC, 50 ACS. Mini International Neuropsychiatric Interview	MDD: 73% TTC vs. 26% ACS. Emotional stress trigger 56% TTC vs. 16% ACS.
El-Sayed et al. 2012 [30]	Case-control study. 24701 TTC, 25069 acute MI. Orthopedic patients. ICD 9CM coding system used.	TTC: anxiety disorders 8.9%; mood disorders 15%. Acute MI: anxiety disorders 3.4%, mood disorders 7.2%.
Compare et al.,2014 [31]	Prospective study. 37 TTC, 37 acute MI, Psychological status was assessed using the PGWBI scale.	TTC patients had a significant increase in levels of psychological distress compared to AMI patients and tend to become more negative over time compared with acute MI patients.

**Figure 8:**
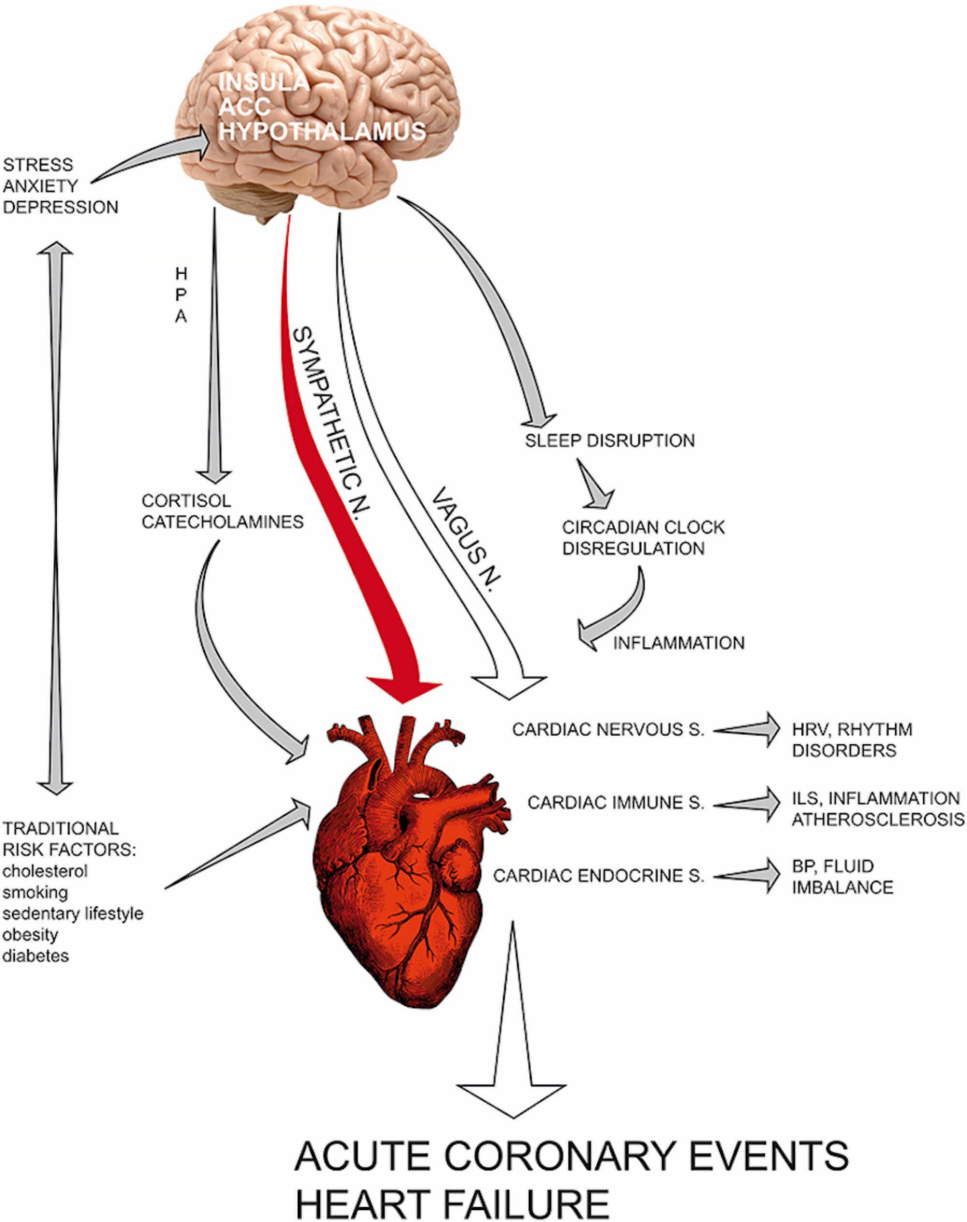
Association of depression and anxiety disorders with different cardiac issues. Source: Fioranelli M, Bottaccioli AG, Bottaccioli F, Bianchi M, Rovesti M, Roccia MG: Stress and inflammation in coronary artery disease: a review. Psychoneuroendocrineimmunology-based. Front Immunol. 2018;9:2031. HPA: hypothalamic-pituitary-adrenal axis; ACC: anterior cingulate cortex; HRV: heart rate variability; ILS: interleukins; BP: blood pressure

There are many psychotherapeutic and psychopharmacological methods available to assist a patient in dealing with stress, including self-help books, counseling services, individual and group psychotherapy, relaxation exercises, and psychotropic medications. Opinions vary on how effective psychotropic medications are in preventing and treating the symptoms of TTC. Some authors doubt they help while others consider them essential. According to various studies, the beta-blockers are used to fight anxiety, especially in postmenopausal women, and can be prescribed for longer periods to control the production of the stress hormones. The main aim of treatment with antidepressants is to relieve the symptoms of depression and anxiety and prevent them from causing TTC (Figure 8) [32].

There is an increased density of postsynaptic b-ARs in MDD. Long-term antidepressant therapy causes the down-regulation of b1-AR (by the inhibition of NE reuptake, stimulation or blockade of receptors, and regulation through serotonergic or dopaminergic systems). Apparently, a paradoxical increase of intracellular cAMP levels was observed at a decreased density of b-ARs, so increased cAMP system activity seems to be fundamental in the therapeutic action of antidepressants. The transcription factor, cAMP response element-binding protein (CREB), is one intracellular target of long-term antidepressant therapy and brain-derived neurotrophic factor (BDNF) is one target gene of CREB. Severe stress leads to a decrease in the expression of BDNF in the hippocampus. According to current theory, long-term antidepressant treatment leads to an increase in the expression of BDNF and his receptor trkB through the elevated function of the serotonin and norepinephrine systems. In theory, through treating the underlying mental illness, it is possible to decrease the probability of cardiac consequences related to high catecholamine levels.

Several studies involving adults with stress-induced cardiomyopathy have not proved that taking commonly used antidepressants lowers the risk of the manifestation and progression of TTC. However, this disease has not been fully studied and thus there are a number of aspects of TTC treatment and prevention that are not yet fully understood.

Takotsubo cardiomyopathy and psychotropic medications: to take or not to take?

According to one study on the relationship of TTC and the use of antidepressants, the syndrome, which is also referred to as stress-induced cardiomyopathy, may follow the same symptoms as those of acute coronary syndrome. In this retrospective descriptive study, 78 patients were involved who met the modified Mayo criteria. Depression and anxiety were diagnosed on the basis of clinical criteria set by a psychiatrist before these patients were admitted to hospital and then clinical outcomes were assessed. Patients having mood disorders may experience an exaggerated response to norepinephrine, leading to an unusually high cardiac sympathetic activity. The researchers showed that there is reduced neuronal norepinephrine reuptake in patients with a major depressive disorder, and this can lead to an increased cardiac sympathetic response [22]. Researchers found that patients taking selective serotonin reuptake inhibitors (SSRIs) had reduced left ventricular ejection fraction (LVEF) recovery after six months and this reduced their chances of survival. SSRIs were recommended for use as antidepressants not because they are more efficacious than other antidepressant agents but because they were associated with fewer side effects. The reduced LVEF and higher patient mortality can be linked with the arrhythmogenic effect of SSRIs and the negative inotropic effect that they exhibit. The results from this study suggested that the administration of SSRIs can increase the probability of inpatient death and, as such, physicians need to consider that these agents have TTC as a possible adverse effect [22].

In one study of TTC caused by selective norepinephrine reuptake inhibitors (SNRIs), researches used a case report of a 52-year-old Hispanic female admitted to the hospital due to TTC caused by venlafaxine. This was a hypertensive patient who was on enalapril 2.5 mg and she had been put on venlafaxine due to her depression a few weeks prior to hospital admission. Echocardiography showed hyperkinesia of the LV basal segment and ballooning of the left ventricle apical segment consistent with the morphology of TTC. Following the diagnosis of TTC, venlafaxine therapy was discontinued and follow-up after six weeks showed a normal LVEF and complete resolution of wall motion abnormalities. Venlafaxine and other SNRIs like duloxetine exert their action by inhibiting the reuptake of catecholamines into the presynaptic neurons; therefore, the probability of a neurotransmitter binding to the postsynaptic neurotransmitter receptors is increased. The postulated mechanism of TTC associated with venlafaxine is by blocking of cardiac fast inwards sodium, and the high synaptic catecholamine levels are responsible for myocardial stunning. Researchers noted that patients with affective disorders have greater cardiac sympathetic sensitivity and this predisposes them to TTC [33]. The findings from the case report in this study support the theory of apical ballooning being related to catecholamines. In this patient, serotonin/norepinephrine reuptake inhibition was responsible for the development of TTC through myocardial stunning. They stated the importance of selectively choosing SNRIs for patients that are at risk of developing TTC. Upon diagnosis of TTC, all patients on SNRIs should have their therapies discontinued and should instead receive other classes of antidepressants. In addition, independent trials evaluating the cardiotoxicity of new SNRI agents should be conducted [33].

In another clinical case presentation, atomoxetine was seen to induce Takotsubo cardiomyopathy. This was a 26-year-old Egyptian woman who had been diagnosed with borderline personality disorder and was put on fluoxetine. From her history, she had unaddressed attention deficit hyperactivity disorder (ADHD) symptoms from the time she was a child and, therefore, atomoxetine 40 mg therapy was initiated and the dose frequency increased thereafter. After four days of therapy, the patient was readmitted following claims of acute central chest pain and dyspnea. An echocardiogram showed basal hyperkinesis and apical kinesis. Serum catecholamines were also elevated. Upon cessation of atomoxetine therapy, echocardiography showed resolution of wall motion and ejection fraction. From this case study, the clinicians considered that the TTC was due to catecholamine-induced coronary vasospasm. Atomoxetine is a substrate for CYP2D6 while fluoxetine is a potent inhibitor. This drug-drug interaction in the patient could explain the high level of atomoxetine and the eventual surge in norepinephrine. Furthermore, the researchers discussed that fluoxetine, through its 5HT2C inhibition, could enhance noradrenergic and dopaminergic activity. Cardiotoxicity associated with atomoxetine was demonstrated from this case [34]. They concluded that clinicians should be cognizant of the likelihood of TTC in patients receiving psychotropic drugs especially those that strongly act on norepinephrine, as well as tricyclic antidepressants (TCA) like imipramine and amitriptyline. Patients might be at a higher risk of developing cardiovascular adverse effects as a result of drug interactions that raise the level of atomoxetine or other agents that have the same pharmacological effect [34].

Duloxetine, an SNRI antidepressant agent, was associated with the development of TTC, according to a study done by Selke et al. [35]. They evaluated a case report of a 59-year-old woman who received duloxetine therapy. TTC, in this case, was characterized by a dynamic electrocardiographic change that is similar to MI but in the absence of obstructive coronary disease and with a left ventricular dysfunction. The patient was on antihypertensive agents and had taken duloxetine two months prior. An upper titration of duloxetine caused the patient to be admitted one week later. Cardiac biomarkers were elevated in this event and ventriculography showed severe left ventricular dysfunction. Following the discontinuation of duloxetine therapy, the plasma levels of catecholamines normalized and the patient's presenting symptoms did not reappear. The multisegmental left ventricular dysfunction resolved as shown by transthoracic echocardiography. According to the authors' adverse probability analysis, it was very likely that an upper titration of duloxetine precipitated TTC in this patient [35].

A report in 2011 was the first to note how the selectivity of duloxetine to norepinephrine translates to adverse clinical effects. Duloxetine and other agents in the SNRI class exert their mechanism of action by inhibiting the neuronal reuptake of norepinephrine, thus increasing its level in the synaptic cleft and binding to postsynaptic receptors. The researchers, therefore, concluded that high levels of norepinephrine result in TTC. They recommended that patients taking agents that act on adrenergic drugs should be screened for TTC before therapy is initiated [35].

Serotonin syndrome has been associated with TTC. Increased synaptic serotonin occurs mostly due to drug interactions, and patients with the syndrome present with autonomic, cognitive, and somatic manifestations. These symptoms can be managed by benzodiazepines and serotonin antagonists in severe cases. A few years ago, clinicians discussed a case of a patient who presented with serotonin syndrome and concomitant stress-induced cardiomyopathy [36]. At the time of presentation, the 46-year-old patient had hyperreflexia with hypertension. A review of her medical history showed that she had used phenethylamine, isocarboxazid, and lithium. This specific combination of three drugs is implicated in serotonergic syndrome. The researchers noted that the patient had concomitant Takotsubo that would rule out the use of a sympathomimetic to manage her hypertension, therefore, this was managed by intravenous fluids. However, atypical antipsychotics like olanzapine and serotonin antagonists have been used to manage severe cases of serotonin syndrome. To their knowledge, this was the first case reported where a serotonin syndrome was linked to the development of stress-induced cardiomyopathy with a reversed TTC pattern. While phenethylamine is known to lead to endogenous catecholamines release, the role of these catecholamines in stress cardiomyopathy is still an area to be studied. There is, however, a possibility of overstimulation of heart serotonin receptors [36]. This group of researchers concluded that the serotonin syndrome in the subject led to physiological stress. In turn, this led to a hyperadrenergic state. The eventual effect was stress-induced cardiomyopathy, a feature of TTC. They recommended further research on the factors that affect individual susceptibility to stress and the role of beta-blockers in preventing the recurrence of serotonin syndrome [36].

In a study of the relationship between cardiogenic shock caused by TTC related to serotonin syndrome, the group of researchers reported a cardiology case of a 65-year-old woman having atrial fibrillation and valvular heart disease, as well as MDD [37]. Echocardiography showed hyperkinesis in the base, akinesis in the apex, and a general ballooning shape, which was part of the diagnosis of TTC in this patient. In patients with stress-induced cardiomyopathy, there is exaggerated sympathetic activation, and serotonin syndrome can lead to high plasma catecholamine levels and trigger the development of TTC in these cases. This patient was on maprotiline, a TCA antidepressant, and dextromethorphan, a cough suppressant. Dextromethorphan is also a 5HT reuptake inhibitor and has been found previously to cause serotonin syndrome. The researchers further noted that the maprotiline and dextromethorphan taken together by their case study patient was the likely cause of serotonin syndrome [37]. Even though the linkage between serotonin syndrome and TTC has not been well-explained, it is likely that the hyperadrenergic state in the syndrome can cause stress-induced cardiomyopathy. There is also a likelihood that the high levels of plasma serotonin might directly overstimulate the heart serotonin receptors. Aside from the serotonin syndrome, the authors explained that improper combinations of antidepressants or their overdose have been shown to influence catecholamines plasma levels and they can lead to the development of TTC. The case, therefore, demonstrated that the concurrent administration of therapeutic levels of maprotiline and dextromethorphan is associated with the development of serotonin syndrome and this can exacerbate TTC [37].

Milnacipran, duloxetine, and venlafaxine are SNRIs that have been approved for the treatment of depression and fibromyalgia in the United States. Milnacipran is well-tolerated in subjects and has an excellent cardiovascular safety profile. In rare occurrences, milnacipran has led to sustained hypertension and tachycardia. In some depressed patients on 150 mg milnacipran, hypertensive crises have been noted. The proposed mechanism of development is increased vascular resistance as a result of higher noradrenergic neurotransmission. In a case study published in 2011, the authors discussed the temporary association of milnacipran use with hypertension, tachycardia, and reversible cardiomyopathy [38]. In their case study, they noted a 42-year-old woman with fibromyalgia who had taken milnacipran to manage it but later developed severe and reversible cardiomyopathy. Tachycardia, hypertension, and high catecholamine levels in plasma were the main signs of a hyperadrenergic state in this patient. The discontinuation of milnacipran therapy and substitution with other agents led to the resolution of cardiomyopathy within six months. Adrenergic blockers and angiotensin-converting enzyme (ACE) inhibitors are used as substitutes in this case. The conclusion from the study was that the patient`s cardiomyopathy was caused by a hyperadrenergic state, and this was in line with previous studies that proved that excess plasma catecholamines led to dilated, hypertrophic cardiomyopathy and regional wall motion defects [38]. All these are symptoms of TTC. Just like the earlier studies, which have been discussed in the above paragraphs, this case study also shows that patients who are on SNRIs like milnacipran should be closely monitored for hypertension and sinus tachycardia as signs of a hyperadrenergic state. Catecholamine-induced cardiomyopathy is reversible upon the discontinuation of the causative SNRIs [38].

A research team carried out a similar study that involved 110 patients. These were prospectively evaluated over six months. Of these, five patients received venlafaxine (n=5) and one received desvenlafaxine. Normetanephrine levels were high in these patients' urine, and echocardiography revealed features like left ventricular dysfunction. The release of normetanephrine is an indication of high levels of catecholamines in plasma. SNRIs increase the plasma levels of catecholamines and the association of TTC with venlafaxine, in this case, is not surprising. SNRIs are prescribed frequently for panic disorders, anxiety, and depression and these patients, therefore, are predisposed to TTC. These researchers, therefore, postulated that the effects of SNRIs in facilitating myocardial exposure to released catecholamines may lead to the development of TTC. SNRIs, therefore, are not a sole precipitant in the development of the disease but they are contributing factors [39].

In another study, a case study of a 43-year-old woman admitted with angina and dyspnea after taking 300 mg venlafaxine was discussed [40]. The authors stated that the woman had taken more than the recommended dose for her condition. A 24-hour urine collection showed high levels of epinephrine while an analysis of magnetic resonance imaging scans showed that there was thickening of the left ventricular wall. Venlafaxine therapy was stopped and replaced with beta-blockers and ACE inhibitors. The findings were similar to related studies on the same topic. The woman in the case study had emotional stress, which led to catecholamine-mediated neurogenic myocardial stunning [40]. However, they did not explain the pathomechanism of the ballooning effect characterized in TTC. After analyzing previous studies on the subject, these researchers noted that the enhanced responsiveness of the apical myocardium to the high levels of catecholamines is what makes it more vulnerable. Venlafaxine is a commonly prescribed antidepressant in the U.S. and has been given to have a safe profile with minor cardiovascular side effects. Many other studies have been done on the potential of venlafaxine causing TTC, along with other SNRIs. A conclusion from the study by Acebes et al., 2011, was that there is a direct association between venlafaxine overdose, high catecholamines levels, and TTC [40].

There is a possible relationship between TTC and tricyclic antidepressants overdose. TTC following nortriptyline overdose was investigated in a study by De Roock [41]. The case report involved a 54-year-old Caucasian woman who had taken an overdose of nortriptyline 1000 mg. Echocardiography revealed apical akinesia, and it was also noted that there was decreased systolic function. Apical ballooning was confirmed by cardiac catheterization and the coronary arteries were seen to be in normal states. Troponin I and CK-MB were at normal levels in this patient. Treatment was by 160 mg aspirin ASA and 5 mg bisoprolol. In this study, the researchers did not elaborate on the exact role that nortriptyline plays in the development of TTC. Plasma catecholamine levels were not measured; this would be employed to give a correlation between norepinephrine levels and QRS duration. They suggested, from previous publications, that nortriptyline reduces coronary flow according to experiments conducted on heart muscles [41].

Prolonged antidepressant therapy can have neuroplastic effects and delay pathological consequences [42]. In post-menopausal women, stress-induced cardiomyopathy is a frequently diagnosed clinical state with dyspnea, angina LV dysfunction, and ECG changes that are similar to myocardial infarction [42]. There was a case of a 65-year-old woman with stress-induced cardiomyopathy after the withdrawal of her antidepressant therapy. The patient was on neuroleptics, SSRIs, TCAs, and benzodiazepines. The rapid withdrawal of SSRI treatment that has been in place for a long time is thought to induce a withdrawal state characterized by psychological and somatic symptoms. The authors stated that this case entailed some rebound in coronary microvascular reactivity. They noted that the latency between stress-induced cardiomyopathy onset and antidepressants discontinuation was unusually long in this case [42].

Lithium carbonate overdoses in bipolar disorder have been linked to TTC according to a case report done by Kitami et al. [43]. In the case report, a 78-year-old woman with a history of hypertension had been ordered lithium 600 mg per day for two years for her bipolar disorder. The presenting symptoms like ataxia, tremor, and myoclonus were linked to severe lithium intoxication and, as a result, this therapy was discontinued. Two days later, the patient developed breathing difficulties and an electrocardiogram showed an elevated ST-segment. Left ventricular dysfunction was seen on echocardiography. Basal hyperkinesia and apical kinesia were characteristic of TTC. The neurologic and cardiac abnormalities disappeared three weeks after the serum lithium concentration was normalized. The authors noted that this was the first documented case of TTC associated with lithium poisoning in bipolar disorder [43]. Lithium, a mood stabilizer, stimulates the production of catecholamines from the adrenal glands in a dose-dependent pattern. In addition, lithium administration can cause catecholamine overload, and this has a special role in the pathogenesis of TTC. Comorbid medical illnesses like cardiac conditions are common in patients having bipolar disease, but it could be coincidental that the patient, in this case, had a cardiac disorder [43].

Neuroleptic malignant syndrome (NMS) is associated with TTC. Kawabata et al., 2003, investigated a case report of a 66-year-old man who had been diagnosed with incomplete NMS due to high levels of muscular enzymes and given hydration therapy [44]. While he was on imipramine and amantadine drugs for the management of his depression and Parkinson's states, the patient had a syncopal attack. Results from an electrocardiogram showed elevated ST, similar to acute myocardial infarction. From an echocardiogram, physicians detected a left ventricular dysfunction and there were hypokinesia and dyskinesis in the anterior and apical wall regions, respectively. The basal wall had hyperkinesis. Despite the patients having normal coronary arteries, the patient's LV contractility had not changed after one month. A perfusion defect was detected, but this resolved after some time. The possible mechanism causing NMS is accelerated norepinephrine function and diminished dopamine function [44]. Even though catecholamine concentrations were not measured in this case, many other cases have reported a high level of catecholamines in TTC. The researchers did not examine the sympathetic nervous system in this case, but its association with NMS is a suggestion that catecholamine abnormality has a role in TTC. The reversible LV dysfunction in the NMS is similar to a stunned myocardium after myocardial infarction. The writers hypothesized, from the case study that coronary microvascular damage caused by disorders of the cardiac sympathetic innervation can lead to TTC. However, these researchers did not give a reason why myocardial damage took place only in the apical part of it [44].

Pharmacologically induced Takotsubo syndrome (PITS) can be used to provide an insight into the mechanisms and treatment of TTC [45]. Murdock et al. conducted an investigation that shows the characteristics shared between PITS and spontaneous TTC. Most of the patients found to have TTC were females of post-menopausal age. In the review, agents used to cause PITS were catecholamines, which led to the activation of beta-adrenergic receptors. The findings from this study are supportive of the hypothesis that catecholamine excess is the primary cause of TTC. Some of the agents used to cause PITs include direct adrenergic agonists, phosphodiesterase inhibitors, vasoconstrictors, and agents like 5-FU [45]. If pharmacologic agents can precipitate TTC independently, it implies that they can synergistically cause TTC in patients that are at risk, more so those that are in hyperadrenergic situations. Stressful events or pain can cause adrenergic states and, in these situations, agents that elevate the level of plasma catecholamines put the patients at risk for TTC. Mostly, the antidepressants of the SSRI and SNRI class are known to elevate plasma levels of catecholamines, but some pain relievers like tramadol also cause this effect [45].

Executive summary

Looking back at all the articles considered for this review, there are possible improvements that could be made to provide readers with a clear clarification on the association between TTC, psychiatric disorders, and antidepressants use. Most of the studies are conducted using small sample sizes. After direct standardization considering the frequency of psychiatric disorder, the association with the use of antidepressants and TTC becomes weak and statistically insignificant. Another potential limitation of the studies has to with age. The studies haven’t investigated the age factor as it supposed to be explored. The researchers ought to have developed a broader scope of age scale to be able to nuance the results to reflect a more thoughtful compilation. At the moment though, the research studies are too generalized on the age perspective. It would have been more interesting to gather which group of women in terms of the age bracket are more vulnerable to TTC and what is the prevalence of different anxiety and depression disorders among women of a particular age. Another perspective that possibly should have been included would have been race and ethnicity. With such an understanding, the studies would have painted a better picture and provided a more nuanced reflection of the manifestation of the condition among women.

The majority of studies mentioned that the subjects received a diagnosis of TTC upon admission to hospital. As opposed to the cases with TTC, in the control group, the diagnosis was based only on past medical history and previous admissions. This approach tends to be flawed, as patients could be undiagnosed for TTC but had initially been screened for the condition. It also limits the frequency of relevant findings. With reduced frequency, it becomes more challenging to quantify results to a myriad of other situations.

The studies show that antidepressants have a critical role in the development of TTC. Most of the case reports were of patients who took overdoses of certain antidepressants or some drug interactions that led to higher levels of catecholamines in plasma. Of great concern is the group of antidepressants that are called SSRIs, like fluoxetine. Another with the same effect are the selective norepinephrine and serotonin reuptake inhibitors (SNRIs) like duloxetine and venlafaxine. It is worthwhile to note that these antidepressants are commonly prescribed in the United States, especially in older patients with other pathological conditions. These agents inhibit the uptake of norepinephrine neurotransmitters to the postsynaptic receptors, leading to unusually higher levels of plasma catecholamine. Post-menopausal women, from the case reports, were found more vulnerable to TTC, probably because of an increased sensitivity of the cardiac cells to catecholamines. Most of these women had physiologic stress, which has a relationship with high hyperadrenergic states. According to the data presented, tricyclic antidepressants must be avoided in patients with heart disease. Prescribers are encouraged to check for drug-drug interactions in patients with more than one medication. The management of drug-induced TTC is through the discontinuation of the current therapy with antidepressants and replacement with beta-adrenergic blockers and ACE inhibitors.

Prior to initiating antidepressant therapy, a comprehensive cardiac history and examination, baseline weight, and vital signs should be obtained. It is real that history should include any signs of cardiac disease in the patient or family, including a history of sudden cardiac death and heart failure. Despite the warning of TTC with antidepressants, there is no enough evidence that antidepressant-treated patients are at a significantly increased risk. If history and physical examination show no significant cardiac disease, routine ECG screening is typically not indicated. According to this data review, antidepressants may still cause and/or exacerbate TTC, and vital signs should be monitored in patients treated with antidepressants.

## Conclusions

In conclusion, the studies discussed here describe Takotsubo cardiomyopathy as a cardiac disorder that affects the left ventricular muscle. It is important to note that the condition can affect anybody of any gender and age. Treatment of the condition is largely management of the symptoms and support of the patients to improve their stress management abilities.

This comprehensive review shows an association between TTC with psychiatric disorders and antidepressants use. The majority of antidepressants are able to block the reuptake of catecholamines, resulting in an accumulation of epinephrine and serotonin in the neuronal synapses and causing significant catecholamine excess, leading to the left ventricular apical kinesis that is similar to the one in acute coronary syndrome. Our article emphasizes the need to explore the interactions between antidepressants use and TTC in studies with an appropriate design and larger sample size. It is extremely important for treating clinicians to obtain a proper history of susceptible patients before antidepressant agents are administered. The use of consulting cardiological services offers us a collaborative team approach when treating patients with TTC.
